# Digital Health Delivery of Parenting Skills to Improve Conduct Problems in Middle School Youth Across Two Distinct Randomized Trials

**DOI:** 10.1007/s11121-024-01750-2

**Published:** 2024-11-18

**Authors:** Elizabeth Stormshak, Arin Connell, Anne Marie Mauricio, Morgan McLaughlin, Allison Caruthers

**Affiliations:** 1 Prevention Science Institute, 6217 University of Oregon, Eugene, OR 97403 USA; 2 Department of Psychological Sciences, Case Western Reserve University, Cleveland, OH 44106 USA

**Keywords:** Parenting skills, Digital health, Middle school, Conduct problems

## Abstract

This study evaluated direct and indirect effects of the Family Check-Up Online (FCU-O) across two distinct randomized trials with middle school youth and families. The FCU-O is a digital health adaptation of the Family Check-Up that is delivered in a brief, online format with supportive coaching. The FCU-O was delivered to parents of middle school youth with a focus on targeting both proximal and distal outcomes that impact the development of conduct problems, including a range of parenting skills, effortful control, and youth emotional problems. Participants were primary caregivers of children aged 10 to 14 years. Eligibility varied across the trials and included endorsing depression or significant stress, or having a child enrolled in a school with limited resources. A total of 374 participants across trials were included in the analysis and were randomly assigned to receive the intervention or control. Using Integrative Data Analytic techniques, we examined the effects of random assignment on a range of outcomes, including emotional and behavior problems, parenting skills, parenting confidence, and effortful control. We examined the indirect effects of the FCU-O on changes in conduct problems from baseline to 6 months via changes in mediator variables at the first follow-up assessment (2–3 months). The FCU-O improved emotional problems, effortful control, parenting confidence, and parent sense of importance at the first follow-up. Mediation analyses suggested indirect effects on conduct problems at 6-month follow-up via improvements in effortful control and emotional problems, but not via improvements in parenting confidence or sense of parenting importance. Results indicate the FCU-O has potential as a public health intervention for families with middle school youth to reduce behavioral risk. ClinicalTrials.gov Identifier: NCT 03060291; NCT 05117099.

**Supplementary Information:**

The online version contains supplementary material available at 10.1007/s11121-024-01750-2.

Although cultures around the world vary in family values, religion, community, and context, the experience of parenting, and being parented, transcends cultures and impacts us all (Bornstein, 2013). Decades of developmental research suggest that warm, supportive, positive parenting skills improve child outcomes, whereas abusive, coercive, neglectful parenting leads to a range of negative outcomes, including behavior problems, mental health disorders, and a lifetime of disrupted relationships (Stormshak et al., 2017). Dr. Robert McMahon and his colleagues set out to improve our science around service delivery and parenting skills interventions over 4 decades ago, applying developmental research to effective intervention delivery for children with behavior problems.

Parenting skills training programs have been well-recognized as highly efficacious treatments for a range of child mental health concerns and are clearly the most effective intervention models for child behavior problems (Shelleby & Shaw, 2014). In a recent Cochrane review, parenting interventions were found to significantly improve behavior problems in children, and costs for service delivery were modest compared to societal costs associated with lifelong mental health disorders (Furlong et al., 2013). The work of Dr. McMahon was foundational to this research and helped to guide both the content and process of effective parenting skills training. In their original book, Forehand and McMahon (1981) specified parenting skills that improve child behavior. This work was significant because it offered a guide for what content should be included in parenting skills interventions. Today, the majority of evidence-based parenting skill interventions include the behavioral skills and concepts outlined in this book. Dr. McMahon’s work was impactful to our understanding of effective interventions for conduct problems and was operationalized in the large-scale multi-site research study in the early 1990s called Fast Track (CPPRG (i.e., Conduct Problems Prevention Research Group [CPPRG], 1992). Findings from the Fast Track study supported parenting skills training as one critical component of a multi-focused prevention approach to reduce problem behavior in young children. Impacts of the Fast Track program on child and adult adjustment were far-reaching, and included improved proximal targets, such as parenting skills and child behavior (CPPRG Group, 1999), as well as reductions in risk behavior across the lifespan, including reduced psychiatric disorders in the adult years (CPPRG, 2011; Dodge et al., 2015).

## The Family Check-Up (FCU) Model

Since Dr. McMahon’s groundbreaking research in the 1990s, youth with histories of adversity, mental health problems, and behavior disorders have continued to be underserved by our schools and mental health systems (Finkelhor et al., 2021). Family interventions that integrate parenting skills training are the key to successful prevention and intervention with children and adolescents at risk of long-term behavioral health problems (McMahon & Pasalich, 2018). The FCU program was inspired in part by McMahon and colleague’s seminal work on school-based prevention and was originally motivated by the goal of developing a family-focused prevention program that could be delivered in schools in a brief, cost-effective manner. The FCU incorporates a strength-based approach to human development that focuses on intervening with individuals in the context of their relationships with family, peers, schools, and communities and is associated with reductions in long-term emotional and behavioral problems (Fosco et al., 2012; Stormshak et al., 2010). In 2015, we created a digital health version of the FCU with the goal of broadening the reach and impact of the intervention in schools. The FCU Online expands on the growing body of research on digital health service delivery over the past 10 years, especially related to family support and intervention. This growth in digital intervention delivery addresses several problems in the field, including access to prevention and intervention in communities with few resources or staffing to deliver programs in person. This is particularly important as mental health access declines, especially for populations that are experiencing health disparity. An increase in online parenting programs occurred over a decade ago, which has allowed for several meta-analyses of outcomes associated with these programs. For example, meta-analyses have examined digital delivery of parenting skills interventions and found significant effects on positive parenting and child problem behavior, as well as parenting confidence and satisfaction (Spencer et al., 2020; McAloon & de la Poer Bereford, 2023). Furthermore, noninferiority analyses of a comparable program, Triple P, suggest equivalency in outcomes associated with digital delivery when compared to in-person treatments for disruptive behavior and parenting skills (Prinz et al., 2022). These intervention targets are consistent with our focus in the FCU Online, which supports both skill development and overall parenting self-efficacy.

In the first randomized clinical trial of the FCU Online (the Middle School Success trial; MSS), we tested the model in an efficacy trial, where it was delivered as a stand-alone program or with coaching by trained research staff (Danaher et al., 2018; Stormshak, et al., 2019). The FCU Online was offered to students across eight middle schools in Oregon (both rural and urban; more than 70% economically disadvantaged), and students were randomly assigned to condition. The FCU Online with coaching improved parents’ self-efficacy (*d* = 0.25) and child emotional problems (*d* = 0.32) at three months post-test, with outcomes moderated by risk in the expected direction (e.g., higher behavioral risk was associated with greater improvements; Stormshak et al., 2019). Furthermore, for children with higher levels of behavior problems at baseline, the FCU Online showed intervention effects on both effortful control and parenting confidence.

In the second randomized trial (the Middle School Success Over Stress trial; MSSOS), we tested the FCU Online model with a sample of middle school children and parents who reported mental health distress after the COVID-19 pandemic. Significant intervention effects were found on both child behavior and parent mental health outcomes (perceived parent stress, emotion regulation, anxiety, and depression). We also observed significant intervention effects for outcomes related to parenting and family relationships, including improvements in positive and proactive parenting as well as reductions in negative parenting and family conflict (Connell & Stormshak, 2023). Parents reported no technology-related barriers and high consumer satisfaction. These data support the acceptability and feasibility of the FCU Online and provide preliminary evidence for its effects on the target mechanisms of change in youth mental health and behavior problems, including parenting skills, family relationships, and effortful control.

## The Present Study

Children with mental health problems are underserved, creating challenges for schools with very limited resources, particularly in today’s post-pandemic era. For example, the National Association for School Psychologists (NASP) recommends a ratio of 1 provider to 500 students, yet estimates nationwide are in the range of 1 provider to over 1000 students (NASP, 2022). In addition, many treatments delivered by schools have limited efficacy and do not address the contexts surrounding mental health distress in youth, such as the family (Garbacz et al., 2018). Collectively, these factors call for the development of sustainable, evidence-based interventions for children and families that school providers with limited time and resources can deliver through existing multi-tiered system of supports (Kearney & Childs, 2021).

Our research focuses on embedding family-centered prevention and intervention in schools to support student mental health and behavior. To date, we have examined outcomes of the FCU Online on parenting skills and effortful control across two distinct clinical trials focused on middle school youth (MSS and MSSOS), but we have not yet integrated findings across these trials or tested the mediating role that effortful control and parenting skills have on long-term conduct problem behavior. The present study extends our work by harmonizing data across MSS and MSSOS using Integrative Data Analytic techniques to examine pooled intervention effects. Potential benefits of these analyses include enhanced statistical power (e.g., to detect moderators or mediating pathways), increased sample heterogeneity (e.g., levels of risk), and the ability to probe possible between-study differences in intervention effects (Curran & Hussong, 2009). We hypothesize that randomization to the FCU Online impacts parenting skills and effortful control, which in turn subsequently reduces behavior problems in youth.

## Methods

### Participants

For both trials in this study, youth and their families were recruited across multiple school districts in the state of Oregon. Demographic data for both trials is shown in Table 1. In the MSS trial, families were randomized within 8 middle schools to receive the FCU Online with coaching, FCU Online only, or school-as-usual. Participants were primary caregivers (*n* = 213) of children in grades 6 and 7, recruited from 8 public middle schools in the Pacific Northwest randomized to FCU Online with coaching or control. The additional families (*n* = 109) who were randomly assigned to the FCU Online only condition were not included in the current study because the MSSOS trial did not include a comparable study arm.

**Table 1:** Sample demographics and patterns of engagement by study

	MSS (*n* = 213)		MSSOS (*n* = 161)	
	*n*	%	n	%
Parent’s gender (women)	198	93	152	94.4
Child’s gender (girls)	100	46.9	83	51.6
Parent’s race/ethnicity				
White/Caucasian	175	82.2	109	67.7
Black/African American	3	1.4	1	0.6
Hispanic/Latinx	9	4.2	30	10.4
Asian/Asian American	1	0.5	3	1.9
Bi/Multiracial	20	9.4	12	7.5
Parent’s education level				
Less than high school degree	4	1.9	6	3.6
High school degree or GED	29	13.6	14	8.7
Partial college	73	34.3	18	11.2
2-year associate’s degree	31	14.6	16	9.9
4-year college degree	45	21.1	57	35.4
Graduate/professional	31	14.6	50	31.1
Child’s grade				
6th	123	57.7	80	49.5
7th	90	42.3	45	28
8th	0	0	36	22.5
Primary caregiver single	53	24.9	28	17.4
Primary caregiver unemployed	13	6.1	11	6.8
Family SES risk (mean, SD)	1.06	1.27	0.99	1.23
*Pattern of engagement, n(%):*				
No app or coach engagement	11	10.2	0	0
App-only	17		1	1.4
Coach-only	0	15.7	1	1.4
App and coach	80	74.1	73	98.6
*Among app engagers, mean (SD):*				
Number of app visits	3.51	− 3.12	10.93	− 4.71
Total time on app (minutes)	62.74	− 61.78	141.91	− 57.65
*Among coaching engagers, mean (SD):*				
Number of coaching sessions	1.83	− 1.23	5.59	− 1.79
Total time with coach (minutes)	44.29	(25.28)	162.49	− 82.67

*MSS* Middle School Success, *MSSOS* Middle School Success over Stress

In the MSSOS trial, families were recruited across the state of Oregon and randomized to the FCU Online with coaching or waitlist control. Participants in the MSSOS trial were primary caregivers (*n* = 161) of children aged 10 to 14 years. Parents qualified for the study if they reported depression or concerns about their child’s adjustment during the pandemic. To be eligible, parents endorsed significant depression on the Patient Health Questionaire-2 (PHQ-2; Löwe et al., 2005) or stress on a 4-item version of the Perceived Stress Scale (PSS; Cohen et al., 1983). Recruiting for the MSSOS trial was initiated following the resumption of school in Oregon after the COVID-19 pandemic (see CONSORT diagram; supplemental material online).

### Procedures

For the MSS trial, families were invited to participate via an e-mail sent to them from their school that explained the study. A research staff member contacted interested parents to further explain the study and provide details about participation. Interested parents were then mailed a packet that included a parent consent form and the surveys. Parents in both conditions completed assessments at baseline, 3 months, and 6 months. For the MSSOS trial, we partnered with schools statewide to recruit families and we invited participation by sending flyers about the study in COVID-19 testing kits, which were sent to families across Oregon as part of a collaborative testing initiative between the University of Oregon and school districts. Families were recruited between October 2021 and March 2022. Interested parents contacted the study team via the project website or a phone call, and a research team member described the study, answered questions, confirmed eligibility, and reviewed the informed consent. Next, parents completed the baseline assessment and were randomized by the research team member to the intervention (with coaching) or waitlist control condition. Parents in both conditions completed baseline, 2-month, 4-month, and 6-month follow-up assessments via an online or telephone survey, per participant’s choice. Across all waves, 97% chose to complete the interviews online.

### FCU-O Intervention Protocol

The FCU-O includes an assessment, computer-generated feedback to guide intervention foci, and intervention modules with content from the *Everyday Parenting Curriculum* (Dishion et al., 2012), including positive parenting, rules and consequences, support for school success, and communication. There were some differences in delivery approach across trials; the MSS trial delivered materials over a web-based platform, while MSSOS delivered materials in an app-based format via smartphones. Additionally, a new module labeled “Healthy Behaviors for Stressful Times” was created for the MSSOS trial, focused on supporting families in the development of healthy routines and structures (i.e., sleep, family activities, and academic support) for coping with stress. Otherwise, intervention modules were generally the same across trials. In both trials, the FCU-O was offered in both English and Spanish, with corresponding telehealth coaching tailored to the parent’s needs.

Parent-coaches in both trials were masters-level clinicians trained to fidelity using the COACH rating system (Smith et al., 2013). Parent-coaches contacted families for an initial meeting to facilitate the use of the FCU-O, set goals, and provide a framework for coaching, including for follow-up coaching meetings after the completion of each FCU-O module using motivational interviewing strategies to engage families in the change process.

### Control Conditions

The MSS trial included an assessment-only control condition. Participants randomized to this condition completed assessments at baseline, 3-, and 6-month follow-up periods but were not offered intervention services through the trial. The MSSOS trial included a waitlist control condition in which participants completed assessments at baseline, 2-, and 4-month assessments before they were provided access to the FCU-O with very limited coaching (e.g., 1 session).

### Demographic Characteristics

Parents completed demographic questions for all family members, including gender, age, race/ethnicity, and indices of Socioeconomic Status (SES). A risk index related to SES was created in both trials based on six dichotomous indicators coded yes/no: high school diploma or GED, the parent is single, income below the poverty threshold (relative to family size), recipient of financial assistance (e.g., food stamps), currently unemployed, and the family’s home is overcrowded (bottom one-third of the sample for room-to-occupant ratio).

### Youth Conduct Problem Behaviors and Emotional Problems

In both trials, parents completed the 5-item conduct problems and emotional problems subscales of the Strengths and Difficulties Questionnaire (SDQ; Goodman, 1997). Items are on a 3-point scale (*0* = *not true to 2* = *certainly true*)*.* For conduct problems, baseline alpha = 0.71 (MSSOS), and 0.68 (MSS). For emotional problems, baseline alpha = 0.74 (MSSOS and MSS).

### Youth Effortful Control Skills

Parents in both trials completed the 8-item youth effortful control skills subscale from the Early Adolescent Temperament Questionnaire (Ellis & Rothbart, 2005), reflecting youth attention and impulse control skills. Baseline alpha = 0.86 (MSSOS) and 0.91 (MSS).

### Parenting Skills and Family Relational Functioning

Parents in both trials completed the Parenting Young Children scale adapted for adolescents (PARYC; McEachern et al., 2012). The current analyses included the limit-setting subscale, with 7-items reflecting setting rules and consequences, baseline alpha = 0.76 (MSSOS) and 0.81 (MSS). We also included two family-relationship subscales: (1) family togetherness, with 3-items reflecting family cohesion (MSSOS alpha at baseline = 0.84; MSS alpha at baseline = 0.85), and (2) family conflict, with 4-items reflecting conflict frequency (MSSOS alpha at baseline = 0.68; MSS alpha at baseline = 0.72).

Parents completed two 14-item subscales adapted from the Parenting Task Checklist (Sanders & Woolley, 2005). Parents responded to items using a 5-point scale (1 = “not at all” to 5 “very”) to indicate how important they viewed each parenting skill, and their confidence in their use of each skill (e.g., “Providing praise and encouragement for good behavior,” and “Applying consequences when rules are broken,”). For confidence, alpha at baseline = 0.88 (MSSOS) and 0.87 (MSS). For sense of importance, baseline alpha = 0.82 (MSSOS) and 0.72 (MSS).

### Data Harmonization

The two trials employed identical measures for core child, parent, and family-level outcomes, and so we employed a logical harmonization strategy to aggregate data across trials. Logical harmonization required that identical scoring approaches were employed across trials for all variables, recoding or rescaling items when necessary to match a common standard across the two samples. Analyses indicated that missing data appeared to be consistent with Missing At Random assumptions in both trials (Connell & Stormshak, 2023; Stormshak et al., 2019).

### Data Analysis

Preliminary analyses examined differences between samples with respect to demographic factors, and baseline levels of scores reflecting child and parent functioning. Correlational analyses examined associations between demographics and baseline levels of treatment targets to identify covariates for intervention-outcome analyses. All hypothesis-testing analyses were conducted in Mplus 8.7 (Muthén & Muthén, 2022). We tested short-term intervention effects at the first follow-up assessment (2 months for MSSOS and 3 months for MSS) with a series of analyses in which the short-term outcome assessment was regressed upon a binary treatment-status variable (0 = waitlist control; 1 = FCU-O), controlling for baseline levels of the outcome, with effect sizes reported as Cohen’s *d* (0.20 = small, 0.50 = medium, 0.80 = large; Cohen, 1988). We initially conducted separate analyses for each outcome variable, and a final model in which all outcomes were included in one model. Results were nearly identical across analyses, and so the final model results are presented.

We examined the indirect effects of the FCU-O on changes in youth conduct problems from baseline to 6 months via changes in mediator variables at the first follow-up assessment to preserve the temporal ordering of parent and youth outcomes. For these analyses, changes in conduct problems were modeled as latent growth models examining the rate of change from baseline to 6-month post-intervention. For latent growth models, we employed the following time-points in MSS (baseline, 3 months, 6 months) and in MSSOS (baseline, 2 months, 4 months, 6 months), with FIML used to account for missing data at time points in each study where assessments were not collected. An example model is shown in Fig. 2 (supplemental resource online). Statistical tests of the indirect effects from intervention to the change in mediator variables at the first follow-up assessment to the linear rate of change in the target outcome were estimated using bootstrapped standard errors to calculate 95% CIs (MacKinnon et al., 2004). We report the Standardized Indirect Effect, which is interpreted as an effect size (Preacher & Kelley, 2011).

All main and indirect-effect analyses controlled for the following covariates: youth gender (0 = cisgender and transgender boys or others including gender nonbinary, 1 = cisgender or transgender girls), youth age (in months), study membership (0 = MSS, 1 = MSSOS), and an SES-risk composite. However, we also re-ran all analyses without controlling for gender, age, or SES-risk and results for both short-term intervention effects and indirect effects were nearly identical. The pattern of significant and non-significant effects was unchanged, and differences in the magnitude of regression estimates were trivial.

## Results

### Preliminary Analyses

#### Descriptive Statistics and Sample Differences

Demographic data is shown in Table 1. Descriptive statistics are shown in Tables A (MSS) and B (MSSOS; supplemental material available online). The MSS and MSSOS trials differed significantly with respect to race/ethnicity (Chi-square [*df* = 6] = 26.21, *p* < 0.001), with the MSSOS trial including a lower percentage of White families, but a higher proportion of Hispanic families. Youth in the MSSOS trial were also slightly older than youth in the MSS trial (*F*(1, 373) = 45.67, *p* < 0.001). However, other demographic factors were not significantly different across studies. We also compared trials on baseline levels of youth, parent, and family functioning outcomes. Significant differences were observed across trials for most variables at baseline, including conduct problems (*F*(1, 373) = 26.35, *p* < 0.001), emotional problems (*F*(1, 373) = 37.08, *p* < 0.001), effortful control skills (*F*(1, 373) = 19.18, *p* < 0.001), limit-setting (*F*(1, 373) = 51.16, *p* < 0.001), and parenting confidence (*F*(1, 373) = 20.68, *p* < 0.001). However, parenting sense of importance did not differ across studies (*F*(1, 373) = 1.57, *p* = 0.21).

#### FCU-O Engagement

Descriptive data regarding patterns of engagement with the FCU-O and parent coaching is shown in Table 1. Differences across studies in terms of categorical patterns of engagement were significant (Chi-square [*df* = 3] = 21.025, *p* < 0.001). Although most parents in both trials engaged with both the online program and coaches (MSS = 74.1%; MSSOS = 97.3%), parents in the MSS trial were more likely to only use the app (MSS = 15.7%) or have no engagement with either the app or coaches (10.2%). Among parents who engaged with the app, those in the MSSOS trial had significantly more visits (*F*(1, 168) = 152.37, *p* < 0.001) and spent significantly more time on the app (*F*(1, 168) = 72.41, *p* < 0.001) compared to those in the MSS trial. Similarly, among parents who engaged with coaches, those in the MSSOS trial had significantly more coaching sessions (*F*(1, 168) = 233.83, *p* < 0.001), and spent significantly more time with coaches (*F*(1, 168) = 148.41, *p* < 0.001) compared to those in the MSS trial.

### Treatment Outcome Analyses

#### Short-Term Intervention Effects

Results for analyses examining the main effects of intervention at the first follow-up assessment are shown in Table 2. For indices of youth functioning, significant short-term intervention effects were observed for reductions in emotional problems (Cohen’s *d* = 0.21) and improvements in effortful control skills (Cohen’s *d* = 0.13). However, there were no significant intervention effects for youth conduct problems (Cohen’s *d* = 0.09). With respect to parental functioning, significant short-term intervention effects were observed for parenting confidence (Cohen’s *d* = 0.19) and sense of parenting importance (Cohen’s *d* = 0.38). However, significant intervention effects were not observed for limit-setting (Cohen’s *d* = 0.13), family conflict (Cohen’s *d* = 0.12) or family togetherness (Cohen’s *d* = 0.14).

**Table 2:** Short-term intervention effects

	Conduct problems		Emotional problems		Effortful control		Limit-setting		Parenting confidence		Parenting importance		Family conflict		Family togetherness	
	β (SE)	*p*	β (SE)	*p*	β (SE)	*p*	β (SE)	*p*	β (SE)	*p*	β (SE)	*p*	β (SE)	*p*	β (SE)	*p*
Intervention assignment	− 0.04 (.03)	*p* = .31	− 0.09 (.04)	*p* = .009	.07 (.03)	*p* = .02	.07 (.05)	*p* = .17	.10 (.04)	*p* = .02	.19 (.04)	*p* < .001	− .04 (.04)	*p* = .30	.08 (.05)	*p* = .08
Baseline-level	0.77 (.02)	*p* < .001	0.75 (.02)	*p* < .001	.82 (.02)	*p* < .001	.49 (.04)	*p* < .001	.66 (.04)	*p* < .001	.57 (.04)	*p* < .001	.67 (.04)	*p* < .001	.54 (.04)	*p* < .001
Baseline covariate effects																
Study-membership	0.26 (.05)	*p* < .001	0.30 (.05)	*p* < .001	− .20 (.05)	*p* < .001	− .33 (.05)	*p* < .001	− .21 (.05)	*p* < .001	− .06 (.05)	*p* = .27	.50 (.04)	*p* < .001	− .31 (.05)	*p* < .001
Youth gender	− 0.14 (.05)	*p* = .004	0.05 (05)	*p* = .28	.19 (05)	*p* < .001	.00 (.05)	*p* = .99	.11 (.05)	*p* = .02	.10 (.05)	*p* = .04	− .07 (.05)	*p* = .11	.10 (.05)	*p* = .04
SES-risk	0.15 (.05)	*p* = .003	0.11 (.05)	*p* = .03	− .16 (.05)	*p* = .002	.04 (.05)	*p* = .43	.13 (.05)	*p* = .01	.15 (.05)	*p* = .003	− .02 (.04)	*p* = .68	.00 (.05)	*p* = .94
Youth age	.04 (.05)	*p* = .46	− .01 (.05)	*p* = .80	− .10 (.05)	*p* = .04	− .07 (.05)	*p* = .19	− .06 (.05)	*p* = .28	− .02 (.05)	*p* = .73	− .09 (.05)	*p* = .07	− .10 (.05)	*p* = .04

Supplemental analyses also included possible treatment x study interactions in order to examine possible differences in intervention effects across the MSS and MSSOS trials. When the interaction effect was significant, follow-up analyses examined intervention effects separately for each trial. Only one significant interaction effect was observed, for parenting sense of importance (estimate = 0.148, *SE* = 0.068, *p* = 0.03). In follow-up analyses, the effect of intervention on change in parenting sense of importance was significant in both samples, but the standardized estimate of the intervention effect was more strongly positive in MSSOS (estimate = 0.268, *SE* = 0.066, *p* < 0.001; Cohen’s *d* = 0.53) compared to MSS (estimate = 0.114, *SE* = 0.055, *p* = 0.04; Cohen’s *d* = 0.22). There were no other significant treatment x study interaction effects.

#### Indirect Effects of Intervention on Conduct Problems Over Time

Analyses of indirect effects of intervention on youth conduct problems at follow-up periods (to 6 months) were limited to variables for which significant short-term intervention effects were observed (emotional problems, effortful control, parenting confidence, and parenting sense of importance). For conduct problems, an unconditional latent growth model including intercept, linear, and quadratic slope parameters provided good fit to the data, (Chi-square [*df* = 7] = 16.22, *p* = 0.023; *RMSEA* = 0.05, *CFI* = 0.99, *TLI* = 0.99, *SRMR* = 0.06), although the residual variance for the quadratic slope was fixed to zero due to a non-significantly negative residual variance.

We extended this model to include covariates, including the focal mediators in four separate analyses (one for each of the variables with significant short-term effects). In the model examining effortful control as a mediator, the linear rate of change in conduct problems was not significantly related to intervention (estimate = − 0.08, *SE* = 0.201, *p* = 0.692). However, the conduct problems slope was significantly related to effortful control skills at the first follow-up assessment (estimate = − 0.644, *SE* = 0.172, *p* < 0.001), which was related to intervention in turn (estimate = 0.071, *SE* = 0.030, *p* = 0.018), controlling for baseline levels of effortful control. The indirect effect of intervention on linear changes in conduct problems via effortful control skills was significant (estimate = − 0.046, 95% CI [− 0.087, − 0.004]).

Similarly, in the model examining mediation via youth emotional problems, the linear rate of change in conduct problems was not related to intervention assignment (estimate = − 0.121, SE = 0.193, *p* = 0.530), but was significantly related to youth emotional problems at the first follow-up assessment (estimate = 0.623, *SE* = 0.167, *p* < 0.001), which was in turn related to intervention status (estimate = − 0.093, *SE* = 0.035, *p* = 0.008), controlling for baseline levels of emotional problems. The indirect effect of intervention on the linear rate of change in youth conduct problems via improvements in youth emotional problems was significant (estimate = − 0.058, 95% CI [− 0.116, − 0.007]).

The linear rate of change in conduct problems was also related to parenting confidence at the first follow-up assessment (estimate = − 0.542, *SE* = 0.202, *p* = 0.007), although it was not directly related to intervention assignment (estimate = − 0.08, SE = 0.224, *p* = 0.719). While parenting confidence was, in turn, significantly related to treatment assignment (estimate = 0.088, *SE* = 0.039, *p* = 0.023), the overall indirect effect of intervention on linear changes in conduct problems via parenting confidence was not significant (estimate = − 0.048, 95% CI [− 0.120, 0.008]). Finally, the linear rate of change in conduct problems was not significantly related to parental sense of importance at the first follow-up assessment (estimate = 0.232, *SE* = 0.252, *p* = 0.357), and the indirect effect was not significant (estimate = 0.044, 95% CI [− 0.078, 0.178]).

## Discussion

This study used an Integrative Data Analytic technique to combine data across two distinct clinical trials that examined the FCU-O delivered in middle school. Both projects collected a range of assessments that were identical across studies, allowing for a logical harmonization strategy. Results suggest direct effects of the FCU-O on a range of proximal outcomes, including effortful control, emotional problems, parenting confidence, and sense of parenting importance. We then examined indirect effects of the intervention on conduct problem behavior 6 months later and found that rate of change in conduct problems was significantly related to effortful control and emotional problems at the first follow-up. The results suggest that short-term impacts on effortful control and emotional problems predicted longer-term changes in conduct problem behavior. These results are promising and highlight the potential of the FCU-O as a digital tool for reducing conduct problem behavior among middle school youth.

Despite overwhelming evidence for the efficacy of parenting interventions, only a small percentage of parents receive this support and most school-based interventions do not integrate family support. One reason for this lack of participation is that parenting training programs are difficult for schools to deliver due, and as a result, uptake of parenting programs in school systems has been moderate to low (Smolkowski et al., 2017). The digital health version of the FCU reduces these barriers and has potential for wide-scale implementation in both healthcare systems and schools. Illustratively, across both trials in this study, a high percentage of parents engaged in the program and connected with a coach (74% in MSS and 98% in MSSOS). These data highlight FCU-O’s potential for wide-scale reach and public health impact when delivered in schools.

There is a critical need to develop and implement digital health interventions that reach families and youth at risk of long-term mental health and behavior problems (Stormshak et al., 2021). Digital health technologies offer significant opportunities for increased access, particularly for families who have traditionally faced difficulties accessing efficacious behavioral healthcare, including racial and ethnic minoritized families and youth who are vulnerable to health disparities (Willis et al., 2022). In addition to increased flexibility and autonomy with respect to time and schedule of participation, digital health technologies are associated with less stigmatization and normalize help-seeking behavior (Metzler et al., 2012), particularly in the current digital context in which engaging in self-help smartphone applications is increasingly normative (Graham et al., 2020). Notably, our results showed that parents in the MSSOS study, compared to those in the MSS study, engaged more with the FCU app and participated in more coaching sessions. In addition to conducting the MSSOS study in a post-pandemic context when behavioral health needs were unprecedentedly high (e.g., Waller et al., 2021), recruitment processes and eligibility criteria also elicited participation from parents and youth with higher levels of psychological distress.

Consistent with differences in eligibility criteria, our preliminary analyses showed that the two samples differed with respect to vulnerability for future mental health disorders, with youth in the MSSOS sample demonstrating more conduct and emotional problems and less effortful control. Moreover, parents in the MSSOS sample reported lower levels of limit-setting and less parenting confidence, which is reasonable given that the MSSOS study was conducted when our nation was still emerging from the pandemic during which parents were presented with a host of novel challenges (e.g., balancing homeschooling with working at home). Despite differences in the two samples, our results for the harmonized sample were robust and demonstrated the efficacy of the FCU-O across families representing significant homogeneity, with intervention effects moderated by study (MSS or MSSOS) for only one outcome, parenting sense of importance (which may have been related to the extended proximity between parents and children during the pandemic, underscoring the salience of parenting). Indeed, an advantage of Integrative Data Analytic methods is the capacity to highlight intervention efficacy among heterogenous samples, which has implications for the targeted reach of an intervention. Illustratively, our study’s results suggest that the FCU-O is feasible and efficacious for families with youth with varying levels of need. Moreover, given heterogeneity across samples with respect to race and ethnicity, our results suggest that the FCU-O is responsive to the needs of culturally diverse families (Mauricio et al., 2021).

### Limitations and Future Directions

Our study demonstrated that the FCU-O worked well for a range of families; however, parents engaged with the product differently based on need. Future research that employs methods such as mixture modeling and mixed designs to examine nuanced differences within and across samples may help more precisely link families with services that are optimal for their family’s unique needs. Our results also suggest that changes in effortful control, not parenting skills, mediated later changes in behavior. These findings are inconsistent with prior studies on the FCU which highlight parenting skills as the primary target linked to changes in youth behavior. More research on the FCU-O and parenting outcomes is needed to fully understand these findings. It may be that changes in parenting confidence, which were observed in this study, lead to changes in parenting skills, which in turn predict later reductions in youth behavior problems. We were unable to test this multi-step mediation model in the current trial due to lack of sufficient follow-up data. Moreover, both trials included in the current harmonization study were FCU-O efficacy trials, with parallel implementation strategies and coaches with comparable background and training. Harmonizing samples across multiple trials of an intervention that differed in implementation strategies could be useful in demonstrating the reliability of intervention effects regardless of implementation differences, or revealing how these implementation differences differentially impact outcomes, informing future Hybrid Type III studies to understand optimal implementation strategies. Additionally, in the MSSOS trial, families in the waitlist condition were provided access to the FCU-O and limited coaching after the 4-month assessment, which may attenuate intervention and control-group differences at the 6-month follow-up in this trial. However, we found significantly reduced engagement with the FCU-O among waitlist families once they received access, and so attenuation would likely be modest (Connell et al., 2024). Further, only two of the four indirect effects that were tested were statistically significant, and corrections for multiple testing were not employed. Caution is warranted in interpreting these mediated pathways, and future replication efforts are needed.

An additional limitation of this study was that data collection for both trials was primarily parent report, which precluded testing outcomes such as parent–child observations or teacher reports of behavior. The limits of self-report may also have impacted our effects on parenting skills, which may be measured more effectively with observation or a multi-rater assessment. Despite our limited effects on skills, parenting confidence and sense of importance were significant outcomes that can be supported through a brief digital program, and improvements in these parenting constructs have the potential to reduce youth behavior problems.

Despite the plethora of data supporting the efficacy of in-person parenting interventions (e.g., Leijten et al., 2019; Sandler et al., 2015), the resources required to implement these interventions with fidelity challenge their feasibility and sustainability. Moreover, research has consistently shown that in-person parenting interventions once translated to real-world practice have extremely poor participation rates, particularly when delivered in schools (e.g., Smolkowski et al., 2017). In summary, the practice and science of parenting interventions have advanced significantly since the seminal text by Forehand and McMahon (1981), progressing from lengthy, in-person, group interventions to the current day self-paced digital interventions with asynchronous delivery and increased access and reach for underserved families.

## Supplementary Information

Below is the link to the electronic supplementary material.

Supplementary file1 (PDF 241 KB)

## Data Availability

Data is available by contacting the first author at bstorm@uoregon.edu.
