# Supplementary material for: Digital Health Delivery of Parenting Skills to Improve Conduct Problems in Middle School Youth Across Two Distinct Randomized Trials
Source: Prev Sci. 2024 Nov 18;26(4):582–91. doi: 10.1007/s11121-024-01750-2 (PMC12208997; doi:10.1007/s11121-024-01750-2)

Digital health delivery of parenting skills to improve conduct problems in middle school youth across two distinct randomized trials

Table A

Descriptive Statistics for Study Outcomes for MSS Trial

	0 months		3 months		6 months		12 months	
	M	SD	M	SD	M	SD	M	SD
Child Functioning								
Conduct Problems	1.24	1.55	1.27	1.54	1.06	1.49	1.14	1.52
Emotional Problems	2.56	2.21	2.39	2.22	2.39	2.22	2.35	2.19
Effortful Control	3.26	.93	3.32	.90	3.34	.87	3.26	.92
Parent Functioning								
Parenting Confidence	4.12	.52	4.12	.59	4.15	.53	4.11	.55
Parenting Importance	4.52	.28	4.52	.30	4.51	.30	4.51	.31
Limit Setting	3.18	.50	3.18	.53	3.15	.52	3.17	.51
Family Relations								
Family Conflict	.83	.78	.76	.73	.79	.68	.72	.72
Family Togetherness	4.00	.81	3.94	.84	3.95	.77	3.89	.84

*Middle School Success (MSS)

Table B

Descriptive Statistics for Study Outcomes for MSSOS Trial

	0 months		2 months		4 months		6 months	
	M	SD	M	SD	M	SD	M	SD
Child Functioning								
Conduct Problems	2.21	2.08	1.85	1.82	1.75	1.87	1.77	1.85
Emotional Problems	4.04	2.46	3.82	2.52	3.51	2.36	3.51	2.49
Effortful Control	2.86	.83	2.94	.81	2.95	.86	2.97	.86
Parenting								
Parenting Confidence	3.86	.57	3.97	.58	3.99	.58	4.01	.65
Parenting Importance	4.48	.38	4.48	.39	4.49	.38	4.46	.48
Limit Setting	2.80	.51	2.89	.51	2.86	.51	2.91	.54
Family Relations								
Family Conflict	1.83	1.09	1.65	1.05	1.51	.98	1.50	1.07
Family Togetherness	3.40	.87	3.49	.88	3.59	.85	3.57	.90

*Middle School Success over Stress (MSSOS)

Digital health delivery of parenting skills to improve conduct problems in middle school youth across two distinct randomized trials

Figure 1

CONSORT Flowchart of Participants

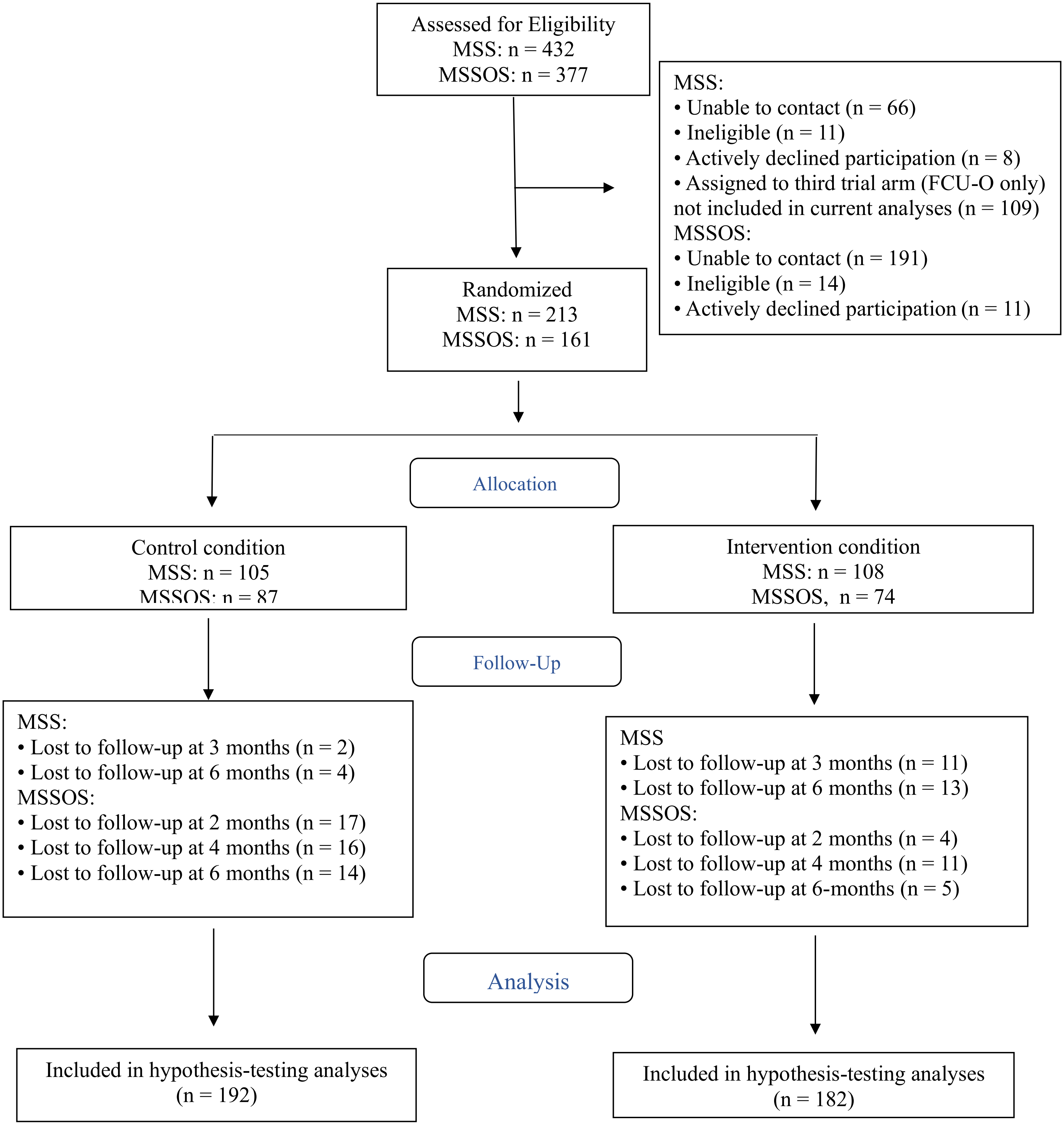

Figure 2 *Latent Growth Model to Examine Indirect Effects of Intervention on Youth Conduct Problems*

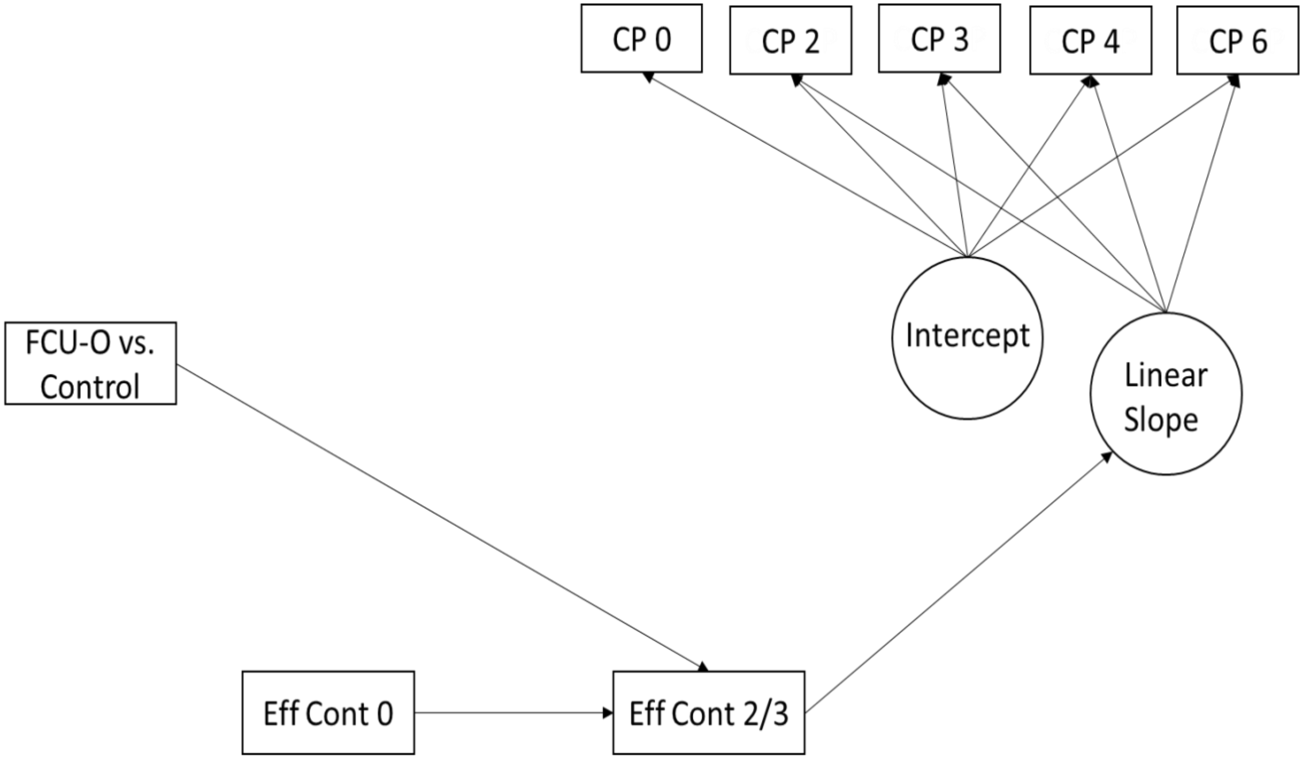

Supplement: Supplementary file 1 — Supplementary file1 (PDF 241 KB) [file 11121_2024_1750_MOESM1_ESM.pdf]
